# Electroacupuncture for Moderate and Severe Benign Prostatic Hyperplasia: A Randomized Controlled Trial

**DOI:** 10.1371/journal.pone.0059449

**Published:** 2013-04-12

**Authors:** Yang Wang, Baoyan Liu, Jinna Yu, Jiani Wu, Jing Wang, Zhishun Liu

**Affiliations:** 1 Guang'anmen Hospital, China Academy of Chinese Medical Sciences, Beijing, China; 2 China Academy of Chinese Medical Sciences, Beijing, China; Northwestern University, United States of America

## Abstract

**Purpose:**

To evaluate the effects of electroacupuncture (EA) on the International Prostate Symptom Score (IPSS), postvoid residual urine (PVR), and maximum urinary flow rate (Qmax), and explore the difference between EA at acupoints and non-acupoints in patients with moderate to severe benign prostate hyperplasia (BPH).

**Subjects and Methods:**

Men with BPH and IPSS ≥8 were enrolled. Participants were randomly allocated to receive EA at acupoint (treatment group, n = 50) and EA at non-acupoint (control group, n = 50). The primary outcome measure includes the change of IPSS at the 6th week and the secondary outcome measures include changes of PVR and Qmax at the 6th week and change of IPSS at the 18th week.

**Results:**

100/192 patients were included. At the 6th week, treatment group patients had a 4.51 (p<0.001) and 4.12 (p<0.001) points greater decline in IPSS than the control group in the intention to treat (ITT) and per-protocol (PP) populations. At the 18th week, a 3.2 points (p = 0.001) greater decline was found in IPSS for the treatment. No significant differences were found between the two groups in Qmax at the 6th week (p = 0.819). No significant difference was observed in PVR (P = 0.35).

**Conclusion:**

Acupoint EA at BL 33 had better effects on IPSS, but no difference on PVR and Qmax as compared with non-acupoint EA. The results indicate that EA is effective in improving patient's quality of life and acupoint may have better therapeutic effects than non-acupoints in acupuncture treatments of BPH.

**Trial Registration:**

ClinicalTrials.gov NCT01218243.

## Introduction

Benign prostate hyperplasia (BPH) is an enlargement of the prostate gland due to progressive hyperplasia of the stromal and glandular cells of the prostate. The prevalence of BPH is as high as 40% in men in their fifties and 90% in men in their eighties [1]. BPH is one of the most common causes of lower urinary tract symptoms (LUTS) which include frequent urination, urgent urination, nocturia, urinary stream hesitancy, straining to void, and dribbling [1]. Although the pathophysiology of BPH is characterized by non-neoplastic histological changes, urine storage and voiding problems increase patients' risk of urinary tract infection and chronic kidney diseases and adversely affect patients' quality of life [2], [3]. Current treatment options for BPH include watchful waiting, lifestyle modifications, alpha blockers, 5 alpha-reductase inhibitors, phytochemicals, and BPH-related surgery [4]. Although most of the aforementioned therapies have various degrees of documented effectiveness in the management of BPH, the use of these interventions are limited to specific patient populations or have certain side effects that interfere with patient's quality of life [5].

Acupuncture is a traditional Chinese medicine treatment which has been commonly used in the management of LUTS in China for thousands of years. The effects of acupuncture on LUTS were well documented in Chinese medicine textbooks and are well-supported by modern research studies [6]. Ricci et al [7] found that electroacupuncture (EA) had better effects in decreasing number of voiding times of urinary urgency that persisted after transurethral resection of the prostate. Kubista et al [8] found that EA could significantly increase the closing pressure in women with stress incontinence as compared with placebo, and Philp et al [9] found that acupuncture increased the bladder capacity in patients with bladder instability. Besides effects on urinary storage problems, acupuncture was also found effective in the prevention of recurrent lower urinary tract infections in adult women [10], [11], in improving the quality of life in patients with chronic prostatis [12], in primary monosymptomatic nocturnal enuresis [13].

BPH is clinically characterized by various LUTS which may include or be similar to urinary urgency, stress incontinence, bladder instability, and UTIs; therefore, we hypothesize that acupuncture may be effective in the management of BPH.

This hypothesis is supported by our previous studies in which we found that acupuncture at BL33 had better effects than terazosin in improving International Prostate Symptom Score (IPSS), post-void residual urine (PVR), and maximum urinary flow rate (Qmax) on patients diagnosed with mild to moderate BPH [14], [15]. In addition, we also compared the therapeutic effectiveness of EA at bilateral acupoints of BL33 with EA at non-acupoints (2 cun [around 6.7 cm] lateral to BL33s) in a randomized controlled pilot study; the results demonstrated acupoint EA was more effective than non-acupoint EA in reducing IPSS [16]. However, terazosin is not necessarily the standardized treatment option for patients with BPH and the pilot study related to effects of acupoint on the EA treatment of BPH has a relative small sample size with efficacy measurements of IPSS only [14]–[16].

Theories of traditional Chinese medicine and results from modern studies indicate that acupoints of the fourteen meridians have specific functional regulatory effect on zang-fu organs [17]–[19]; however, studies in western countries found that dry needling, an acupuncture procedure at trigger points (including non-acupoints that do not belong to the meridian system), were effective in the management of various diseases [20]–[21]. Both dry needling and traditional acupuncture treat diseases via inserting stainless needles into the human body. However, differences between acupuncture at acupoints and acupuncture at non-acupoints have not been fully investigated. In the present study, we aimed to evaluate the effects of EA on IPSS, PVR, and Qmax, and explore the difference between EA at acupoints and non-acupoints in patients with moderate to severe BPH.

## Methods

The protocol for this trial and supporting CONSORT checklist are available as supporting information; see Checklist S1 and Protocol S1.

### Study Design

This was a randomized control trial which was performed at the Acupuncture Department of Guang An Men Hospital, China Academy of Chinese Medical Sciences. The study protocol was registered with ClinicalTrials. gov (Identifier: NCT01218243) and was previously published [22]. In the original study protocol, patients with IPSS score higher than 20 (including 20) were proposed to be excluded; however, we changed the inclusion criteria to patients with IPSS≥8 during the actual study, as most patients who visited the acupuncture department and were willing to participate in the study had moderate to severe BPH. The study design complies with the Consolidated Standards of Reporting Trials (CONSORT) and was approved by the hospital ethics committee. Participants were recruited through advertisements on local newspapers and posters and signed informed consent before study participation.

Sequentially numbered, opaque, sealed envelopes were distributed to patients by an investigator who was not involved in acupuncture procedures and data analyses. Based on odd or even numbers assigned in the envelope, participants were randomly allocated to receive EA at acupoint (treatment group, n = 50) and EA at non-acupoint(control group, n = 50).

Based on the results of our pilot study, the reduction of IPSS of the EA at acupointgroup was seven [16]. Therefore, in conjunction with methods and results of the studies by Chapple et al [23] and Yu et al [24], 50 patients will be needed for each group (1:1 allocation) in the present study to detect a seven point reduction in IPSS with a two-tailed significance level of 5% and a power of 90% while allowing for a 20% dropout rate. Sample size was calculated with a standard deviation (SD) of 2.30 as per reports in the epidemiology monograph by Wang [25].

### Participants

For inclusion, the following criteria were fulfilled: 1) 50–70 years old; 2) mild to moderate BPH evaluated by I-PSS; 3) patients having urinary dysfunction more than 3 months; 4) patients with stable life signs; 5) not on any α1 receptor blocker, 5α-reductase inhibitor or traditional Chinese medicine for over 1 week; 6) volunteer to join this research and give informed consent prior to receiving treatment. For safety reasons, patients were instructed of possible emergency conditions and were told to seek appropriate medical help if happens.

The exclusion criteria included 1) urinary dysfunction caused by gonorrhea or urinary tract infection; 2) oliguria and anuria caused by urinary calculi, prostate cancer, bladder tumor and acute/chronic renal failure; 3) urinary dysfunction caused by neurogenic bladder, bladder neck fibrosis and urethral stricture; 4) failure of invasive therapy for prostatic obstruction; 5) injured local organs, muscle and nerve caused by pelvic operation or trauma; 6) upper urinary obstruction and hydrocoele combined with damaged renal function due to BPH diagnosed by B-ultrasound; 7) Patients unable to commit to treatment because of commuting problems to the hospital.

Initial diagnosis and assessment were made by an urologist who was not involved in the study. Patients on LUTS medications were requested to stop medications one week before baseline assessment. The baseline assessments included IPSS, PVR and Qmax.

### Acupuncture protocol

Huatuo brand needles (size 0.30 mm×100 mm, manufactured by Suzhou Medical Appliance, Suzhou, Jiangsu Province, China) together with GB6805-2 Electro-Acu Stimulators (HuayiMedical Supply &Equipment Co., Ltd, Shanghai, China) were used. In traditional Chinese medicine, acupuncture at Baliao points (BL31-34) are commonly used as treatment options for LUTS, lumbodynia, and reproductive disorders [6]; our previous study confirmed the effects of acupuncture at BL33 on LUTS [14]–[16]. In the present study, BL33s were used in the acupoint treatment group; whereas the two points which are 2 cun (around 6.7 cm) lateral to BL33s were used in the non-acupoint control group. Localization of BL33 which corresponds with the third sacral foramen was reported previously [26]. In order to accuratelylocalize bilateral BL33, a line is drawn between the two posterior superior iliac spines (PSIS) which cross over at point A of the spinal column. Then, each PSIS and point A are connected to construct two separate lines. Finally, two different equilateral triangles are drawn inferiorly and the apexes B and C of these two equilateral triangles are the two BL33 acupoints. The acupuncturists inserted acupuncture needles at BL33obliquely at an angle of about 45° for about 6 cm–8 cm until the patient felt heaviness and numbness locally or even with radiation sensation to the genitalia. Acupuncture procedures were performed bilaterally at two BL33 acupoints and electrodes of the electric stimulator were attached to the handle of the needle bilaterally. Disperse-dense wave, 20 Hz electric current was used in the present study. The intensity of electric current was increased to the patients' maximum tolerance and then slightly reduced to a bearable level. Acupuncture treatment protocol and electric stimulator parameters were the same for non-acupoint acupuncture procedures in the control group, except for the genitalial radiating sensation upon acupuncture.

Patients received a total of 16 sessions of acupuncture treatment which include five sessions in the 1^st^ and 2^nd^ week, acupuncture once a day; three sessions in the 3^rd^ and 4^th^ week, acupuncture every other day. Acupuncture procedures were implemented by an acupuncturist with more than 10 years' clinical experience. Data management and analysis were performed by researchers who were blinded to the acupuncture procedures.

### Outcome Assessment

All patients were evaluated during the first week for baseline values which include IPSS, PVR, and Qmax. The primary outcome involves the change of IPSS at the 6th week; secondary outcomes include the changes of PVR, Qmax at the 6th week and change of IPSS at the 18th week. Safety evaluation includes hematoma, fainting, severe pain, and local infection during and after acupuncture. In addition, emergency conditions which require catheterization were also recorded if any.

### Statistical analysis

The statistical analysis was performed by a statistician blinded to treatment allocation in the Clinical Evaluation Center of China Academy of Chinese Medical Sciences. SPSS statistical package program (ver.16.0) was used and a significance level was set at α<0.05. Data analysis of baseline characteristics was based on the intention-to-treat (ITT) population which included all participants who were randomized. Primary and secondary outcomes were mainly based on the data of the ITT population; however, primary outcome was also analyzed based on per-protocol (PP) population as an extra supportive analysis. Quantitative data of IPSS and Qmax were expressed with mean±SD; PVR was expresses as Range (Median) as the data was not normally distributed. For primary and secondary outcome measures, analysis of covariance was used to investigate the differences between acupoint acupuncture and non-acupoint acupuncture on IPSS and Qmax. Changes of the IPSS and Qmax after treatment were the dependent variable, the group was the fixed factor and the baseline data were the covariate. The Mann Whitney U test was used for the analysis of PVR.

## Results

**Figure 1:**
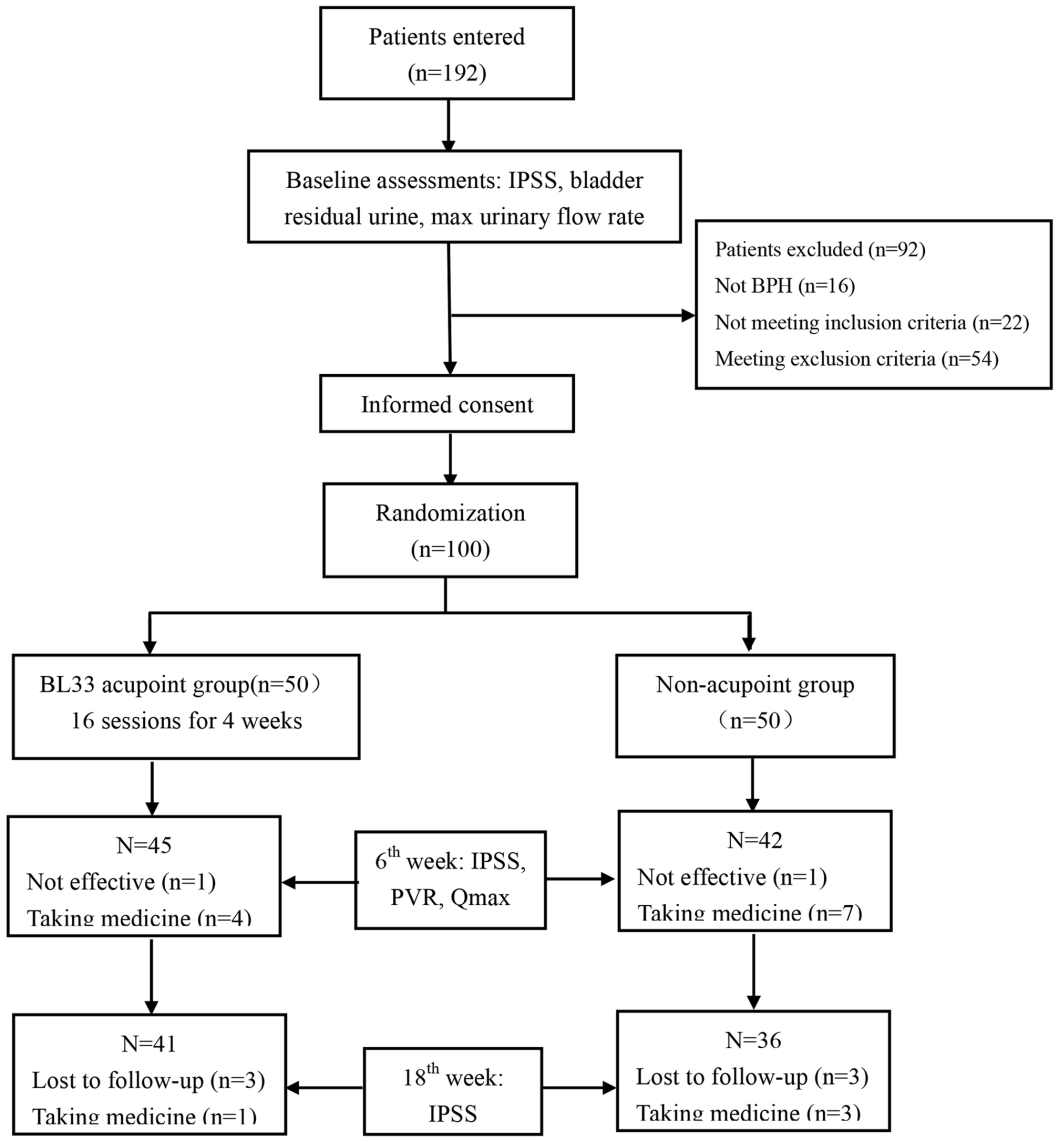
The flowchart of study participation.

**Figure 2:**
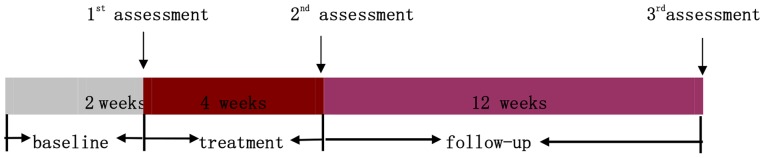
Time frame of each period. Figure 2 shows the time frame of baseline period, treatment period and follow-up period.

From September, 2010 to May, 2012, a total of 192 patients with LUTS visited the Acupuncture Department at Guang'anmen Hospital in Beijing. 92 patients were excluded from the present study for the following reasons: 16 did not have BPH; 22 did not meet the inclusion criteria; 54 met the exclusion criteria. 100 of them were included and equally randomized to receive acupoint acupuncture and non-acupoint acupuncture treatments (Figure 1). Figure 2 details the time frames of recruitment, treatment and follow-up periods.

Demographic characteristics and baseline information of the 100 participants are shown in Table 1. 25 patients in the acupoint acupuncture group and 23 in the non-acupoint control group were diagnosed as severe BPH (IPSS≥20). No statistically significant differences were found between the two groups in age, gender, and baseline values. The mean age of all participants was 65.37±6.89 years old.

**Table 1:** Demographic Information and Baseline Characteristics.

	Acupoint group (n = 50)	Non-point group (n = 50)	P-value (two-tailed)
Age	64.80±7.05	65.94±6.74	0.411
Course of disease	76.08±57.59	73.66±57.72	0.834
IPSS	20.10±6.52	18.76±6.06	0.289
Qmax	13.04±6.73	15.93±7.33	0.051
PVR (ml)	20 (0,128)	16 (0,128)	0.260

### IPSS

Analyses of IPSS at the 6th week were based on both of ITT population and PP population (See Table 2 and Table 3). At the 6^th^ week, the ITT analysis indicated that IPSS reduced from 20.10±6.52 at baseline to 12.84±5.87 for the acupoint treatment group, and from 18.76±6.06 at baseline to 16.42±6.80 for non-point control group. With the PP analysis, IPSS of the two groups reduced to 12.60±5.85 and 16.05±6.83 respectively. At the 6th week, acupoint group patients had a 4.51 (p<0.001) and 4.12 (p<0.001) points greater decline than the non-acupoint control group in the ITT and PP populations respectively (Table 4). At the 18th week, a 3.2 points (p = 0.001) greater decline was found for the acupoint treatment group as compared with the non-acupoint control group.

**Table 2:** Descriptive Statistics of ITT Population.

		Acupoint group (n = 50)	Non-point group (n = 50)
IPSS	baseline	20.10±6.52	18.76±6.06
	6^th^ week	12.84±5.87	16.42±6.80
	Change in IPSS	7.26±5.12	2.34±4.85
	18^th^ week	14.62±5.76	16.96±6.47
	Change in IPSS	5.48±5.16	1.8±5.06
PVR (ml)	Baseline	20 (0,128)	16 (0,128)
	6^th^ week	20 (0,300)	15(0,180)
	Change in PVR	0 (−172,84)	0 (−120,80)
Qmax,	Baseline	13.04±6.73	15.93±7.33
	6^th^ week	12.63±6.11	15.00±6.50
	Change in Qmax	0.36±4.51	0.98±4.05

**Table 3:** Descriptive statistics of PP population.

		Acupoint group (n = 45)	Non-point group (n = 42)
IPSS	Baseline	19.84±6.46	18.83±6.00
	6^th^ week	12.60±5.85	16.05±6.83
	Change in IPSS	7.24±5.23	2.79±5.17

**Table 4:** ANCOVA estimation of outcomes.

		Treatment effect estimate (Mean difference)	Standard error	P-value
IPSS (ITT)	6^th^ week	4.51	0.93	0.000
	18^th^ week	3.20	0.93	0.001
IPSS (PP)	6^th^ week	4.12	1.03	0.000
Qmax(ITT)	6^th^ week	0.18	0.80	0.819

ANCOVA: Analysis of Covariance.

### Qmax and PVR

No significant differences were found between the two groups in Qmax at the 6th week (p = 0.819, Table 4). PVR data followed a non-normal distribution and no significant difference was found (P = 0.35).

### Adverse Events

No serious adverse events happened in eithergroups. Two cases of mild hematoma were reported in the non-acupoint control group during study. The patients were told to apply ice and compression within 24 hours and heat compression after 24 hours to the acupuncture treatment areas. Hematoma disappeared in about two weeks.

## Discussion

The results of this trial showed that greater decrease in IPSS in the BL33 group than in the non-acupoint group, but no significant difference was found in Qmax and PVR. The change of IPSS indicates that EA at acupoints significantly improved the quality of life in patients diagnosed with BPH. The IPSS change matched the results of our previous studies and added further credence in the use of acupuncture for patients with BPH [14]–[16]. The IPSS was decreased by 7.26 (from20.10 to 12.84, P = 0.000) and 2.34 (from 18.76 to 16.42, P = 0.001) in BL33 acupoint EA group and the non-point EA group respectively. Compared to the non-point control group, the acupoint group was associated with a 4.51-point greater decline in IPSS at the 6th week (P<0.01). The difference of IPSS decrease indicates that EA at acupoints had significantly better effects than EA at non-acupoint on the quality of life in patients diagnosed with BPH. As EA procedures at both acupoint and non-acupoint share the same electric stimulation parameters and same EA protocol, the specificity of needling site at BL33 acupoint may be accountable for the difference in IPSS in the present study. This echoed the results of our pilot study stating that acupoints have better effects than non-acupoints [16]. The results of the present study also increases the credibility of results of other related studies indicating that the acupoint of the meridian system seem to have specific functional regulatory effects compared to non-acupoints [17]–[19].

In a similar trial comparing EA with sham EA (shallow needle insertion of 2 mm) for BPH, greater increase of Qmax was found in the EA group than the sham EA group; whereas IPSS was similar in both groups [27]. With a close analysis, acupoints use, manipulation methods, parameters of EA and number, frequency and duration of treatment sessions could all cause the differences between the two studies. Nonetheless, both studies demonstrated that standard EA at acupoint had better effects than EA with shallow needle insertion of 2 mm or EA at non-acupoints on at least certain clinical parameter(s). In addition, IPSS improve in the present study is also supported by results from the study by Johnstone et al [28] in which some difference in IPSS (p = 0.063) was found between acupuncture and blank control. As only 20 out of 30 patients were treated with acupuncture in the study by Johnstone et al [28], the p value is likely to drop to lower than 0.05 if the sample size is increased. Consequently, we should believe the specificity of acupoints in acupuncture treatment even though the mechanism of acupuncture at acupoints has not been fully elucidated.

Studies demonstrate that sacral neuromodulation could improve symptoms of overactive bladder [29]–[30]. As BL33 is actually the third sacral nerve which travels through the 3^rd^ sacral foramen, we should believe that EA at BL33 is actually one type of sacral neuromodulation. Sacral acupuncture was found to be effective in improving symptoms of acetic acid-induced bladder irritation in rats through inhibition of capsaicin-sensitive C-fiber activation [31]. Therefore, EA for BPH may be related to acupuncture sacral neuromododulation.

Although the exact mechanism of EA treatment for BPH has yet to be clarified, researchers believe that the effects of acupuncture are less likely to be related to histological changes of the prostate, as no difference was found in PSA levels, between sham and vera EA and between acupuncture and blank control [27], [28]. As results of acupuncture fMRI studies showed a significant connection between brain activities and acupuncture procedures, and the human brain is closely involved in the sensation and control of the lower urinary system [32]–[34], we should also believe that brain modulation by acupuncture may also play a role in the effects of EA on BPH. This was confirmed by in-vivo animal study in which Chung et al [35] found that expression of c-Fos expression in the pontine micturition center (PMC), ventrolateral periaqueductal gray (vlPAG), and medial preoptic nucleus (MPA) was increased in stress urinary incontinence, and acupuncture significantly decreased c-Fos expression in these areas. Nonetheless, further studies are needed to explore the mechanism of acupuncture on BPH.

### Limitations

As blinding is difficult in acupuncture studies, real randomized placebo controlled trials seem impossible. Although non-acupoint EA procedures were used as control in the present study, they are still acupuncture procedures; thus we could not rule out the confounding factor of needling and placebo effects in the present study. Patients in the present study were only treated for 4 weeks and followed up till the 18th week, long term effects and optimal treatment regimens of EA for BPH remain to be established. In addition, this RCT was performed in only one hospital rather than multi-centers; therefore, the results of the present study may not well-characterize the general response of patients with BPH in the world. To further test the therapeutic effects of EA on BPH, further large scale, multi-center, international cooperative studies are warranted.

## Conclusion

In the present study, acupoint EA at BL 33 had better effects on IPSS, but no difference on PVR and Qmax as compared with non-acupoint EA. The results indicate that EA is effective in improving patient's quality of life and acupoint may have better therapeutic effects than non-acupoints in acupuncture treatments of BPH.

## Supporting Information

Checklist S1 CONSORT Checklist.
(PDF)

Protocol S1 Trial Protocol.
(PDF)
