# Supplementary material for: Electroacupuncture for Moderate and Severe Benign Prostatic Hyperplasia: A Randomized Controlled Trial
Source: PLoS One. 2013 Apr 12;8(4):e59449. doi: 10.1371/journal.pone.0059449 (PMC3625218; doi:10.1371/journal.pone.0059449)
Supplement: Checklist S1 — CONSORT Checklist. (PDF) [file pone.0059449.s001.pdf]

Table 1. STRICTA 2010 checklist of information to include when reporting interventions in a clinical trial of acupuncture.

Item	Detail	Section
**1. Acupuncture rationale**	1a) Style of acupuncture (e.g. Traditional Chinese Medicine, Japanese, Korean, Western medical, Five Element, ear acupuncture, etc)	Method
	1b) Reasoning for treatment provided, based on historical context, literature sources, and/or consensus methods, with references where appropriate	Introduction
	1c) Extent to which treatment was varied	no
**2. Details of needling**	2a) Number of needle insertions per subject per session (mean and range where relevant)	Method
	2b) Names (or location if no standard name) of points used (uni/bilateral)	Method
	2c) Depth of insertion, based on a specified unit of measurement, or on a particular tissue level	Method
	2d) Response sought (e.g. *de qi* or muscle twitch response)	Method
	2e) Needle stimulation (e.g. manual, electrical)	Method
	2f) Needle retention time	Method
	2g) Needle type (diameter, length, and manufacturer or material)	Method
**3. Treatment regimen**	3a) Number of treatment sessions	Method
	3b) Frequency and duration of treatment sessions	Method
**4. Other components of treatment**	4a) Details of other interventions administered to the acupuncture group (e.g. moxibustion, cupping, herbs, exercises, lifestyle advice)	Method
	4b) Setting and context of treatment, including instructions to practitioners, and information and explanations to patients	Introduction
**5. Practitioner background**	5) Description of participating acupuncturists (qualification or professional affiliation, years in acupuncture practice, other relevant experience)	Method
**6. Control or comparator interventions**	6a) Rationale for the control or comparator in the context of the research question, with sources that justify this choice	Discussion
	6b) Precise description of the control or comparator. If sham acupuncture or any other type of acupuncture-like control is used, provide details as for Items 1 to 3 above.	Method
