# Supplementary material for: Electroacupuncture for Moderate and Severe Benign Prostatic Hyperplasia: A Randomized Controlled Trial
Source: PLoS One. 2013 Apr 12;8(4):e59449. doi: 10.1371/journal.pone.0059449 (PMC3625218; doi:10.1371/journal.pone.0059449)
Supplement: Protocol S1 — Trial Protocol. (PDF) [file pone.0059449.s002.pdf]

Wang et al. Trials 2011, 12:211
http://www.trialsjournal.com/content/12/1/211

TRIALS

STUDY PROTOCOL Open Access

Efficacy of electroacupuncture at Zhongliao point (BL33) for mild and moderate benign prostatic hyperplasia: study protocol for a randomized controlled trial

Yang Wang, Zhishun Liu*, Jinna Yu, Yulong Ding and Xuan Liu

Abstract

Background: Acu-point specificity is a key issue in acupuncture. To date there has not been any satisfactory trial which can ratify the specific effect of acupuncture. This trial will evaluate the specific effect of BL33 for mild and moderate benign prostatic hyperplasia (BPH) on the basis of its effectiveness. The non-specific effect will be excluded and the therapeutic effect will be evaluated.

Method: This is a double-blinded randomized controlled trial. 100 Patients will be randomly allocated into the treatment group (n = 50) and the control group (n = 50). The treatment group receives needling at BL33 and the control group receives needling at non-point. The needling depth, angle, direction, achievement of *De Qi* and parameters of electroacupuncture are exactly the same in both groups. The primary outcome measure is reduction of international prostate symptom score (IPSS) at the 6th week and the secondary outcome measures are reduction of bladder residual urine, increase in maximum urinary flow rate at the 6th week and reduction of IPSS at the 18th week.

Discussion: This trial will assess the specific therapeutic effect of electroacupuncture at BL33 for mild and moderate BPH.

Trial registration: Protocol Registration System of Clinical Trials.gov NCT01218243

Keywords: Electroacupuncture, Acu-point Efficacy, BPH, RCT

1. Background

For thousands of years, points of the fourteen meridians have always maintained their specific locations, and therapeutic indications. Point specificity is the central part of acupuncture and plays an important role in clinical practice. It has been proved by Cochrane reviews that acupuncture is effective for migraine prophylaxis, tension headache and chemotherapy-induced nausea or vomiting [1-3]. But there still remains a level of uncertainty about the specificity of some acupuncture ponits [4]. The difficulty in resolving this problem is due to the fact that acupuncture outcome is influenced by complex interplay of insertion depth, angle, and achievement of *De Qi*. Recently published trials in Germany show that acupuncture was no more effective than sham acupuncture for migraine, headache and knee osteoarthritis [5-8]. One could attribute it to adenosine's role in the mechanistic actions of acupuncture. According to the latest research [9], ATP release from keratinocytes in response to insertion and manual rotation of acupuncture needles results in an accumulation of adenosine that transiently reduces pain. If relief of pain is purely a response to mechanical stimulation, then it does not matter much which acupuncture point is being used. Hence, it is not appropriate to study point specificity with pain syndromes.

Study on point specificity began in China in the 1980s and it has been found that points of the fourteen

* Correspondence: liuzhishun@yahoo.com.cn
Acupuncture Department, Guang'anmen Hospital Affiliated to China Academy of Chinese Medical Sciences, No.5 Beixiange Street, Xuanwu District, Beijing, China

BioMed Central

© 2011 Wang et al; licensee BioMed Central Ltd. This is an Open Access article distributed under the terms of the Creative Commons Attribution License (http://creativecommons.org/licenses/by/2.0), which permits unrestricted use, distribution, and reproduction in any medium, provided the original work is properly cited.

meridians have specific functional regulatory effect on zang-fu organs [10-12], such as St36's specific effect for visceralgia and SI19's specific relationship with the ear. According to the results of those studies and our clinical practice, we took a trial showing that electroacupuncture at BL33 can reduce IPSS by 6.52 ± 0.41 (n = 47) [13] and 6.68 ± 2.84 (n = 87) [14]and improve the symptoms of difficult urination in mild or moderate patients with BPH. Its effect is better than that of terazosin. Also in our pilot trial (n = 40), 40 patients were randomized into the treatment group (BL33) and the control group (non-point acupuncture site located beside BL33). The result shows that electroacupuncture at BL33 has better effect in reducing IPSS and bladder residual urine and increasing maximum urinary flow rate than electroacupuncture at non-point. There is significant improvement with urinary frequency and nocturia in the treatment group as compared with the control group [15].

This trial is a large-scale prospective randomized trial after the pilot study. Patients and assessors are blinded with regard to acupuncture treatment given. It will compare needling at BL33 with needling at non- point. The same manipulating methods (needling depth, angle, direction and achievement of *De Qi,.*) could cause the same needle sensation between the two groups. As all points used in the trial are on the back area where patients are unable to see, thus blinding for the patients could be applied successfully. The study is registered with an identifier (NCT01218243) by Clinical Trials.gov in USA.

2. Methods and Design

2.1 Design

This study is a prospective randomized controlled trial and will be completed in Acupuncture Department of Guang'anmen Hospital affiliated to China Academy of Chinese Medical Sciences from Sep. 2010 to July 2012. Randomization is performed by Drug Clinical Trial Office affiliated to Guang'anmen Hospital. Computer-made opaque sealed envelopes are numbered consecutively. All of the envelopes are preserved by an investigator (who is uninvolved in acupuncture operation and data analysis). After the baseline assessment has been carried out and the informed consent has been obtained, an envelope is opened by the investigator according to patient's sequence entering the trial and the allocated treatment regimen is offered to the clinician. Two copies of the envelopes will be kept to guard against researchers trying to change the randomization.

Participants are recruited from PBH clinic in the hospital by the use of local newspaper advertisements and posted notices at clinic site. Patients on mediciation for urinary symptoms are requested to stop medication one week before baseline assessment. Before randomization, patients will complete 1-week baseline assessment which includes IPSS, bladder residual urine and maximum urinary flow rate and they will be divided into three groups according to their IPSS score, i.e. the mild group (0-7), moderate group (8-19) and the severe group (20-35). Patients belonging to the severe group will be excluded.

Enrolled patients will be randomized into the treatment group or the control group. The diagnosis and assessment will be made by an independent urologist who is uninvolved in the treatment process. Acupuncture will be given 5 sessions/week in the first two weeks and 3 sessions/week in the third and the fourth weeks, a total of 16 sessions.

The trial protocol is in accordance with the principles of the Declaration of Helsinki and has been approved by the review board and ethics committee of the hospital. Participants will be requested to sign an informed consent before any treatment is given. All patients have right to decide whether they would sign up or not and they can withdraw from the trial at any time (Figure 1).

Patients

According to our 40-case pilot trial and study of Christopher R. Chapple and Hong-Jeng Yu [16,17], to detect a reduction in IPSS of 7 with a two-sided 5% significance level and a power of 90%, 50 patients will be needed for treatment group and control group respectively (1:1 allocation) allowing for a 20% dropout rate [18].

$$n = n1 = n2 = (U\alpha + U\beta)^2 \times (1 + 1/k)\, s^2/\delta^2$$

Inclusion criteria

According to the diagnosis recommended by the fifth international BPH Consulting Committee, patients who meet the following criteria will be recruited: 1) 50-70 years old; 2) mild to moderate BPH evaluated by I-PSS; 3) patients having urinary dysfunction more than 3 months; 4) patients with stable life signs; 5) no use of $\alpha 1$ receptor blocker, 5α-reductase inhibitor or traditional Chinese medicine for over 1 week; 6) volunteer to join this research and give informed consent prior to receiving treatment.

Exclusion criteria

Patients with any of the following conditions will be excluded: 1) urinary dysfunction caused by gonorrhea or urinary tract infection; 2) oliguria and anuria caused by urinary calculi, prostate cancer, bladder tumor and acute/chronic renal failure; 3) urinary dysfunction caused by neurogenic bladder, bladder neck fibrotic and urethral stricture; 4) failure of invasive therapy for prostatic obstruction; 5) injured local organs, muscle and

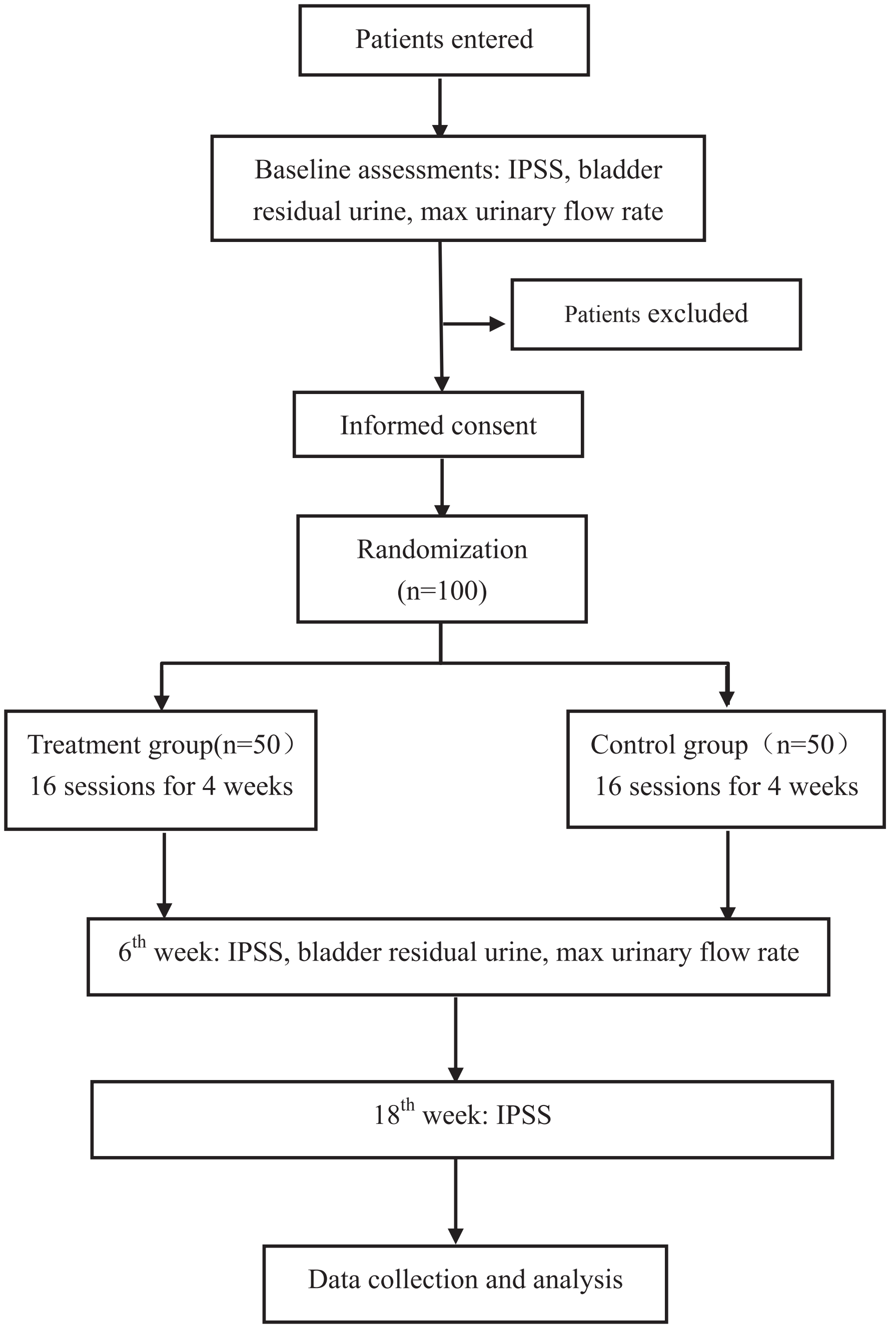

Figure 1 Flow chart of the trial.

nerve caused by pelvic operation or trauma; 6) upper urinary obstruction and hydrocele combined with damaged renal function due to BPH diagnosed by B-ultrasound; 7) Patients unable to commit to treatment because of commuting problems to the hospital.

Interventions

In addition to records in ancient books [19], we also choose this acupuncture point according to our published study [13,14] and a consensus with acupuncture experts in Beijing. Huatuo Brand needle (0.3*100 mm, Suzhou Medical Appliance Factory CL) and G6805-2 electric stimulator (Shanghai Huayi Medical Instrument Co.Ltd) will be used in the trial.

Treatment group

Needle at bilateral BL33 with a 45°angle. A feeling of soreness and distension will be felt when needling into the 3rd posterior sacral foramina (S3) and finally with

radiation of sensation to the perineum. Needle 60-80 mm without lifting, thrusting or rotating. Put on the electric stimulator with a disperse-dense wave, 20Hz. The current intensity is increased to the patients' maximum tolerance and then slightly reduced to a bearable level.

Control group

Take the site 2 *cun* lateral BL33 as the non-point (Figure 2) for the control group. Manipulation methods and electric stimulator parameters are the same with those of the treatment group. The pilot study shows that patients in the control group have the same feeling with those of the treatment group.

There are 16 sessions for all patients (5 sessions in the 1st and 2nd weeks, 3 sessions in the 3rd and 4th weeks) and each session last 30 minutes. Acupuncture for the two groups will be operated by the same acupuncturist with more than ten years of experience. This acupuncturist is blinded to the outcome assessment at baseline, the 6th week and the 18th week.

Outcome measures

The primary outcome measure is reduction of international prostate symptom score (IPSS) at the 6th week compared with baseline. The secondary outcome measures are reduction of bladder residual urine, increase in maximum urinary flow rate at the 6th week and reduction of IPSS at the 18th week.

Safety evaluation is based on adverse events which include hematoma, fainting, severe pain, and local infection. Any adverse event and the time and measures to take will be recorded in detail during treatment.

Statistical analysis

The statistical analysis will be performed by a statistician blinded to allocation in Clinical Evaluation Center of China Academy of Chinese Medical Sciences. Statistical analyses will be performed using the SPSS statistical package program (ver.16.0) and the level of significance will be established at $\alpha < 0.05$. The data analysis of baseline characteristics is based on the intention-to-treat (ITT) population (data of all participants who are randomized will be analyzed). The data analysis of the primary and secondary outcomes is mainly based on the ITT population. In addition, the data analysis of the primary outcome is also based on per-protocol (PP) population as a supportive analysis.

For primary and secondary outcome measures, analysis of covariance will be used to investigate whether BL33 acupuncture is more effective than non-acupoint acupuncture.

Quality control

Before the trial, all staff involved will be trained in patients selection and exclusion, filling up of the case report and acupuncture method. During the trial, supervisors will check on case reports, and acupuncture operation twice a month. Drop-outs, withdrawals (and the reasons), the compliance of all patients will be recorded in detail throughout the treatment and follow-up period.

Discussion

The point specificity has been a controversial research topic for a long time [20] and it has become a point of

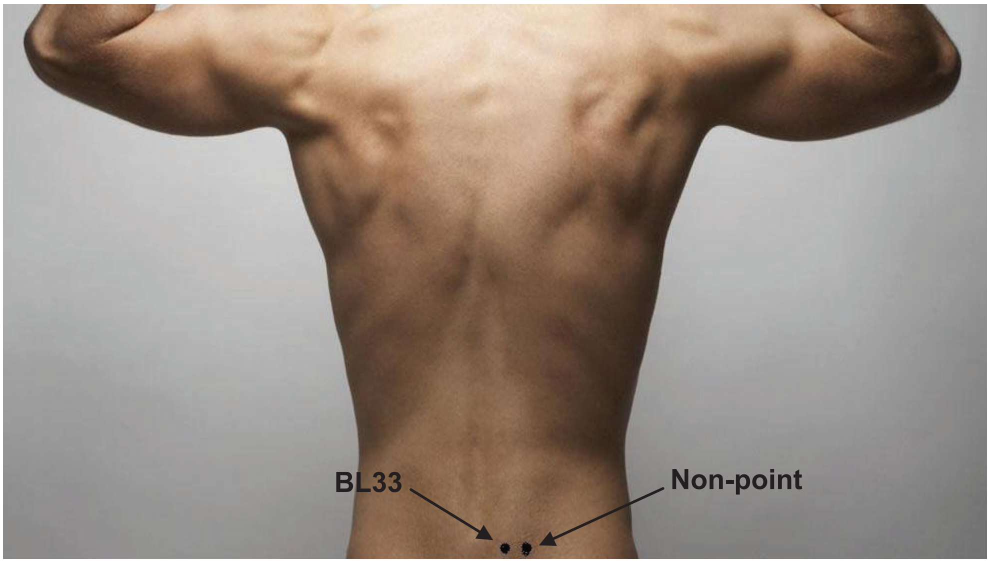

Figure 2 BL33 and non-point used in the trial.

contention since the publication of those trials [5-8] in Germany as previously mentioned. But from the viewpoint of Nanna Goldman [9], anti-nociceptive effect of acupuncture is a general reaction to the mechanical stimulation leading to the belief that pain syndrome should not be used in point specificity study at the present time.

The reasons we choose BPH for point specificity include: 1) studies show that acupoints have specific effect in regulating function of zang-fu organs [10-12] and BPH is appropriate for this kind of study; 2) our previous study shows that electroacupuncture has effect in improving the symptoms of BPH and the effect is better than that of non-acupoint acupuncture; 3) a single point acupuncture is most suited for point specificity study.

In this trial, points used here are on the back area where patients unable to see and patients of the two groups have the same feeling because the manipulation methods are all the same. So patients are blinded as to which treatment they will receive. As the manipulation methods of the two groups are the same, the different effects between the two groups will be completely produced by point location, which will give a good explanation of point specificity. And also the placebo effect could be excluded and the specific effect of electroacupuncture at BL33 could be investigated. As electroacupuncture is also used in the control group with the same parameter and same insertion depth, part of treatment effect could be achieved. So part of specific effect of the treatment group will be excluded inevitably (Figure 3).

This study will follow rigorous methodological requirements. The staffs in charge of assessment and statistician are all blinded to the patients' allocation. Acupuncture operation(performed by an experienced acupuncturist)and filling of case report (by a postgraduate) form will be done under strict supervision. Phone calls and e-mails will be used to inform the patients for follow-up assessment which will be done at their homes or in the clinic. Any medicine taken and other treatment conducted during this time will be recorded in detail.

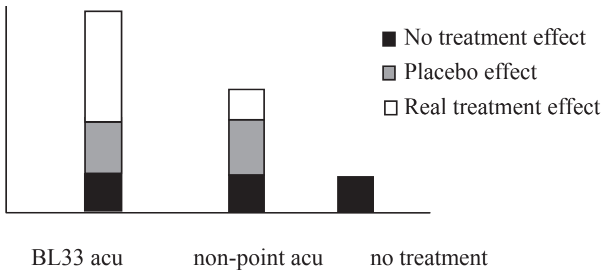

Figure 3 Effects of BL33 acupuncture and non-point acupuncture of this trial.

Conclusion

The aim of this study is to investigate whether BL33 acupuncture is more effective than non-acupoint acupuncture, i.e. point specificity of BL33 for BPH.

Acknowledgements

Written consent for publication was obtained from the patients or their relatives.

Authors' contributions

ZL conceived of the study, and participated in its design and coordination. JY, YD and YW participated in the design of the study. YW and XL drafted the manuscript. All authors read and approved the final manuscript.

Competing interests

The authors declare that they have no competing interests.

Received: 10 August 2011 Accepted: 26 September 2011
Published: 26 September 2011

References

1. Linde K, Allais G, Brinkhaus B, *et al*: **Acupuncture for migraine prophylaxis.** *Cochrane Database Syst Rev* 2009 (issue 1).
2. Linde K, Allais G, Brinkhaus B, *et al*: **Acupuncture for tension-type headache.** *Cochrane Database Syst Rev* 2009 (issue 1).
3. Ezzo JM, Richardson MA, Vickers A, *et al*: **Acupuncture-point stimulation for chemotherapy-induced nausea or vomiting.** *Cochrane Database Syst Rev* 2006 (issue 2).
4. Campbell A: **Point specificity of acupuncture in the light of recent clinical and imaging studies.** *ACUPUNCTURE IN MEDICINE* 2006, **24(3)**:118-122.
5. Linde K, Streng A, Jurgens S, Hoppe A, Brinkhaus B, Witt C, *et al*: **Acupuncture for patients with migraine: a randomized controlled trial.** *JAMA* 2005, **293(17)**:2118-25.
6. Melchart D, Streng A, Hoppe A, Brinkhaus B, Witt C, Wagenpfeil S, *et al*: **Acupuncture in patients with tension-type headache: randomised controlled trial.** *BMJ* 2005, **331(7513)**:376-82.
7. Witt C, Brinkhaus B, Jena S, Linde K, Streng A, Wagenpfeil S, *et al*: **Acupuncture in patients with osteoarthritis of the knee: a randomised trial.** *Lancet* 2005, **366(9480)**:136-43.
8. Scharf H-P, Mansmann U, Streitberger K, Witte S, Kramer J, Maier C, *et al*: **Acupuncture and knee osteoarthritis: a three-armed randomized trial.** *Ann Intern Med* 2006, **145(1)**:12-20.
9. Goldman N, Chen M, Fujita T, *et al*: **Adenosine A1 receptors mediate local anti-nociceptive effects of acupuncture.** *Nature neuroscience* 2010, **13(7)**:883-888.
10. Shuran J, Zhongsuan H: **Specific effect of acupuncture at ST36 for visceral pain.** *Acupuncture Reaearch* 1987, **1**:73-76.
11. Cheng K, Han Y: **Study on the Specificity of Effects of Acupuncture of Different Acupoints in Reducing Gentamycin Induced Ototoxicity in Guinea-pigs.** *Acupuncture Reaearch* 2004, **29(3)**:204-208.
12. Xu B, Yu X, Chen C, *et al*: **Relationship Between Efficacy of Electroacupuncture and Electroacupuncture Stimulation of Different Acupoints and Different Tissue Layers of Acupoint Area in Hypotension plus Bradycardia Rats.** *Acupuncture Reaearch* 2010, **35(6)**:422-428.
13. Yang T, Zhang XQ, Feng YW, *et al*: **Efficacy of Electroacupuncture in Treating 93 Patients with Benign Prostatic Hyperplasia.** *Chinese Journal of Integrated Traditional and Western Medicine* 2008, **28(11)**:998-1000.
14. Yang T, Liu Z, Zhang XQ, *et al*: **Evaluation on therapeutic effects of electro acupuncture for benign prostatic hyperplasia: A prospective randomized controlled study.** *Chinese Journal of Rehabilitation Medicine* 2008, **23(11)**:1028-1031.
15. Ding Y, Yu J, Liu Z: **A pilot trial of point speciality using electroacupuncture at Zhongliao point for benign prostatic hyperplasia.** *Journal of Clinical Acupuncture and Moxibustion* .
16. Chapple RC, Montorsi F, Teuvo LJT, *et al*: **Silodosin therapy for lower urinary tract symptoms in men with Suspected Benign Prostatic Hyperplasia: Results of an International, Randomized, Double-Blind,**

Placebo- and Active-Controlled Clinical Trial Performed in Europe. *European Urology* 2011, **59**:342-352.
17. Yu HJ, Lin A, Yang SD, *et al*: **Non-inferiority of silodosin to tamsulosin in treating patients with lower urinary tract symptoms (LUTS) associated with benign prostatic hyperplasia (BPH).** *BJU INTERNATIONAL* 2011, doi: 10.1111/j.1464-410X.2011.10233.x.
18. Wang J: **Clinical Epidemiology-design, measurement and evaluation.** Shanghai: Shanghai Science and Technology Press;, 2 2001, 141-142.
19. Xie H: *Huangdi Neijing* Puslishing House of Ancient chinese medical books; 2001.
20. Li L, Qin W, Bai L, *et al*: **Exploring vision-related acupuncture point specificity with multivoxel pattern analysis.** *Magnetic Resonance Imaging* 2010, **28(3)**:380-387.

doi:10.1186/1745-6215-12-211
Cite this article as: Wang *et al*.: Efficacy of electroacupuncture at Zhongliao point (BL33) for mild and moderate benign prostatic hyperplasia: study protocol for a randomized controlled trial. *Trials* 2011 **12**:211.

Submit your next manuscript to BioMed Central and take full advantage of:

- **Convenient online submission**
- **Thorough peer review**
- **No space constraints or color figure charges**
- **Immediate publication on acceptance**
- **Inclusion in PubMed, CAS, Scopus and Google Scholar**
- **Research which is freely available for redistribution**

Submit your manuscript at
www.biomedcentral.com/submit

BioMed Central
